# Predicting Early Viral Control under Direct-Acting Antiviral Therapy for Chronic Hepatitis C Virus Using Pretreatment Immunological Markers

**DOI:** 10.3389/fimmu.2018.00146

**Published:** 2018-02-07

**Authors:** James A. Hutchinson, Kilian Weigand, Akinbami Adenugba, Katharina Kronenberg, Jan Haarer, Florian Zeman, Paloma Riquelme, Matthias Hornung, Norbert Ahrens, Hans J. Schlitt, Edward K. Geissler, Jens M. Werner

**Affiliations:** ^1^Department of Surgery, University Hospital Regensburg, Regensburg, Germany; ^2^Department of Internal Medicine I, University Hospital Regensburg, Regensburg, Germany; ^3^Center for Clinical Studies, University Hospital Regensburg, Regensburg, Germany; ^4^Department of Transfusion Medicine, University Hospital Regensburg, Regensburg, Germany

**Keywords:** hepatitis C virus, direct-acting antiviral therapy, immune monitoring, biomarker, memory T cell, non-classical monocyte, classifier

## Abstract

Recent introduction of all-oral direct-acting antiviral (DAA) treatment has revolutionized care of patients with chronic hepatitis C virus (HCV) infection. Regrettably, the high cost of DAA treatment is burdensome for healthcare systems and may be prohibitive for some patients who would otherwise benefit. Understanding how patient-related factors influence individual responses to DAA treatment may lead to more efficient prescribing. In this observational study, patients with chronic HCV infection were comprehensively monitored by flow cytometry to identify pretreatment immunological variables that predicted HCV RNA negativity within 4 weeks of commencing DAA treatment. Twenty-three patients [genotype 1a (*n* = 10), 1b (*n* = 9), and 3 (*n* = 4)] were treated with daclatasvir plus sofosbuvir (SOF) (*n* = 15), ledipasvir plus SOF (*n* = 4), or ritonavir-boosted paritaprevir, ombitasvir, and dasabuvir (*n* = 4). DAA treatment most prominently altered the distribution of CD8^+^ memory T cell subsets. Knowing only pretreatment frequencies of CD3^+^ and naive CD8^+^ T cells allowed correct classification of 83% of patients as “fast” (HCV RNA-negative by 4 weeks) or “slow” responders. In a prospective cohort, these parameters correctly classified 90% of patients. Slow responders exhibited higher frequencies of CD3^+^ T cells, CD8^+^ T_EM_ cells, and CD5^high^ CD27^−^ CD57^+^ CD8^+^ chronically activated T cells, which is attributed to bystander hyperactivation of virus-non-specific CD8^+^ T cells. Taken together, non-specific, systemic CD8^+^ T cell activation predicted a longer time to viral clearance. This discovery allows pretreatment identification of individuals who may not require a full 12-week course of DAA therapy; in turn, this could lead to individualized prescribing and more efficient resource allocation.

## Introduction

The recent introduction of all-oral direct-acting antiviral (DAA) therapy has revolutionized care of patients with chronic hepatitis C virus (HCV) infection. Unfortunately, the very high cost of these drugs limits the access to therapy for many patients and has now become the primary barrier to HCV eradication (1, 2). Sofosbuvir (SOF) is currently priced at $1,000 per day, and SOF with ledipasvir (LDV) at $1,125 a day. Total treatment costs can be as high as $150,000 per patient. With more than a million patients needing HCV treatment in the next 3–5 years in the USA alone, the exceptional prices of these drugs will substantially impact healthcare budgets (3). Owing to the tolerability of DAA treatment and rarity of virological breakthrough, response-guided therapy is currently not recommended for any of the approved DAA regimens (4); therefore, most patients are currently still treated for a fixed duration of 12 weeks (5). Nevertheless, there is considerable variation between patients in time to stable HCV RNA negativity under DAA treatment. If patients who respond fastest to treatment could be identified then, an individually tailored, more cost-effective approach to prescribing shorter courses of DAA therapy may be possible; in turn, this could lead to more efficient resource allocation and treatment of more patients.

Direct-acting antiviral agents fall into four classes defined by the steps in the life cycle of HCV that they disrupt—namely, NS3/4A protease inhibitors (e.g., paritaprevir), NS5B nucleoside polymerase inhibitors (e.g., SOF), NS5B non-nucleoside polymerase inhibitors (e.g., dasabuvir), and NS5A inhibitors [e.g., LDV, daclatasvir (DCV)]. These drugs are administered in combinations chosen on the basis of viral genotype (GT) and residual liver function. Following joint guidelines from the American Association for the Study of Liver Diseases and the Infectious Diseases Society of America (6), patients with GT1 (4/5/6) infection without cirrhosis should be treated for 12 weeks with either a combination of LDV and SOF, or a combination of ritonavir-boosted paritaprevir, ombitasvir, and dasabuvir, optionally with ribavirin (RBV). Alternatively, DCV may be used in combination with SOF for 12 weeks. Patients with GT2 infection should be treated with SOF and RBV for 12 weeks or, like patients with GT3 infection, they could be treated with a combination of DCV and SOF for 12 weeks (7). Currently, the only exception to 12-week courses of treatment is a recommendation made in the European Association of the Study of the Liver guidelines that DAA therapy may be shortened to 8 weeks in previously untreated patients with GT1 infection who are without cirrhosis and whose baseline HCV RNA level is below 6 million international units per milliliter (8). From a health-economics standpoint, it would be valuable if other subgroups of patients who only require short-course DAA therapy could be easily and reliably identified.

Over the last decades, it has been established that chronic antigen stimulation during persistent infection with HCV is associated with continuous activation and impaired function of several immune cell populations such as natural killer cells and CD8 T cells (9). Virus-specific T cell responses are skewed in the chronic phase of HCV infection, due to viral escape and functional exhaustion of virus-specific CD4^+^ and CD8^+^ T cells (10). Consequently, remaining antigen-specific T cells show increased expression of inhibitory molecules such as programmed death-1 and 2B4 (CD244) (11). Accordingly, in this study, patients receiving DAA treatment were comprehensively monitored to identify immunologically related biomarkers from peripheral blood that accurately predicted early HCV RNA negativity. This led to a predictive model based on CD3^+^ T cell and naive CD8^+^ T cell frequencies that correctly classified patients as “fast” responders (i.e., HCV RNA negative within 4 weeks of commencing DAA therapy) in 90% of cases.

## Materials and Methods

### Clinical Study

Peripheral blood samples were provided by patients with chronic HCV infection, who were participating in an observational trial (clinicaltrials.gov: NCT02904603) authorized by the local ethics committee (votum: 14-101-0049). From all participating patients, an informed written consent was obtained. For the training set, 23 patients [GT 1a (*n* = 10), 1b (*n* = 9) and 3 (*n* = 4)] were treated with DCV/SOF (*n* = 15), LDV/SOF (*n* = 4), or with ritonavir-boosted paritaprevir, ombitasvir, and dasabuvir (*n* = 4). A prospective validation set of 10 patients were treated with DCV/SOF (*n* = 1), LDV/SOF (*n* = 7), SOF/RBV (*n* = 1), or LDV/SOF/RBV (*n* = 1). Serum HCV RNA was quantified using the Abbott RealTime HCV assay (Abbott Molecular, Des Plaines, IL, USA) with a lower limit of detection and quantification of 12 IU/ml.

### Flow Cytometry

Whole blood samples were collected in EDTA tubes by peripheral venepuncture and then immediately delivered to the laboratory at ambient temperature. Preanalytical samples were stored at 4°C until processing began within 4 h. Whole blood was stained with DuraClone Immuno-monitoring panels (Duraclone IM phenotyping BASIC Tube, B53309; Duraclone IM T cell Subsets Tube, B53328; Duraclone IM TCRs Tube, B53340; Duraclone IM Treg Tube, B53346; Duraclone IM B cells Tube, B53318; Duraclone IM Dendritic Cell Tube, B53351; all from Beckman Coulter, Krefeld, Germany) according to the manufacturer’s recommendations or antibodies listed in Table 1. Data were recorded with a Navios™ cytometer running Cytometry List Mode Data Acquisition and Analysis Software for Navios™ Cytometer, version 1.3 from Beckman Coulter. Blinded analyses were performed using Kaluza version 1.3 by an experienced operator and verified by a second experienced operator.

**Table 1:** A consolidated flow cytometry panel.

	488 Excitation					633 Excitation			405 Excitation	
	FL1 (FITC)	FL2 (PE)	FL3 (ECD)	FL4 (7-AAD)	FL5 (PE-Cy7)	FL6 (APC)	FL7 (AF700)	FL8 (APC-AF750)	FL9 (Pac-Blue)	FL10 (Aqua)
Antigen	CD45RA	CD5	CD27	Live-Dead	CCR7	CD4	CD8	CD3	CD57	CD45
Clone name	2H4	BL1a	1A4CD27	Optional	G043H7	13B8.2	B9.11	UCHT1	NC1	J.33
Isotype	mIgG1	mIgG2a	mIgG1		mIgG2a	mIgG1	mIgG1	mIgG1	mIgM	mIgG1
Amount (μl)	8	20	8	+	10	6	2	8	8	8
Supplier	BC	BC	BC	BC	BC	BC	BC	BC	BC	BC
Cat. #	6603904	A07753	B26603	A07704	B46025	IM2468	B49181	A94680	A74779	B36294
Status	RUO	CE	ASR	CE	ASR	CE	CE	CE	ASR	CE

*FITC, fluorescein isothiocyanate; PE, phycoerythrin; ECD, phycoerythrin-texas red; 7-AAD, 7-aminoactinomycin D; PE-Cy7, phycoerythrin-cyanin 7; APC, allophycocyanin; AF700, alexa fluor 700; APC-AF750, allophycocyanin alexa fluor 700; Pac-Blue, pacific blue; BC = Beckman Coulter*.

### Statistics

Principal component analysis (PCA), receiver operator characteristic curve analysis, and binary logistic regression were performed in SPSS^®^ release 23.0.0.0 (IBM Analytics, New York, NY, USA). GraphPad Prism 6.04 (GraphPad Software, Inc., La Jolla, CA, USA) was used for chi-squared and Kruskal–Wallis tests, as well as generating plots.

## Results

### Immunological Markers Affected by DAA Treatment

A sustained virological response to DAA therapy is generally achieved in 85–99% of chronic HCV patients, depending upon viral GT, patient-related factors, and the choice of treatment regime (8). In the present study of 23 patients with chronic HCV infection, 22 had achieved a sustained virological response at 12 weeks post-treatment (SVR12) (Supplement S1 in Supplementary Material). Clearance of virus was associated with improved liver function and reduction in systemic markers of liver inflammation (Supplement S2 in Supplementary Material). Although most patients achieved SVR12, there was considerable variation in time to HCV RNA negativity and normalization of biochemical parameters. Baseline mean HCV RNA titer was 6.12 ± 0.12 log_10_ IU/ml, falling to 1.2 ± 0.14 log_10_ IU/ml by 4 weeks, at which time 12/23 patients were HCV RNA < 12 IU/ml. HCV establishes persistent infections during which virus-specific CD4^+^ and CD8^+^ T cells become impaired through progressive functional exhaustion or clonal deletion, and intrahepatic NK cell responses are subdued (12). The objective of this study was to explain variation in 4-week response rates to DAA therapy in terms of immunological factors inherent to patients before treatment. In particular, this study aimed to identify a minimal set of immunological markers that accurately classified patients with chronic HCV infections as “fast responders” (i.e., HCV RNA < 12 IU/ml by 4 weeks) or “slow responders” (i.e., HCV RNA ≥ 12 IU/ml at 4 weeks, irrespective of later outcome). Ideally, these biomarkers were to be independent of viral GT, baseline fibrosis score, previous HCV treatment, and other patient-related factors (Supplement S3 in Supplementary Material) (13).

Significant advances in standardization of multiparametric flow cytometry methods for detecting peripheral leukocyte populations have been made over the last 5 years (14, 15). Following standardized flow cytometry protocols developed by The ONE Study consortium (www.onestudy.org), we measured frequencies and absolute counts of 79 predefined leukocyte subsets in serial whole blood samples. Baseline samples were taken immediately before starting DAA treatment, followed by sampling at 4, 12, and 24 weeks after start of therapy. Our immunophenotyping strategy gave a high-resolution picture of all major blood leukocyte subsets, including their activation or maturation status. A blinded operator evaluated all flow cytometry data and analyses were checked by a second blinded operator. From these results, we assembled information about 79 parameters from 23 patients sampled at 4 visits into a single dataset.

**Figure 1:**
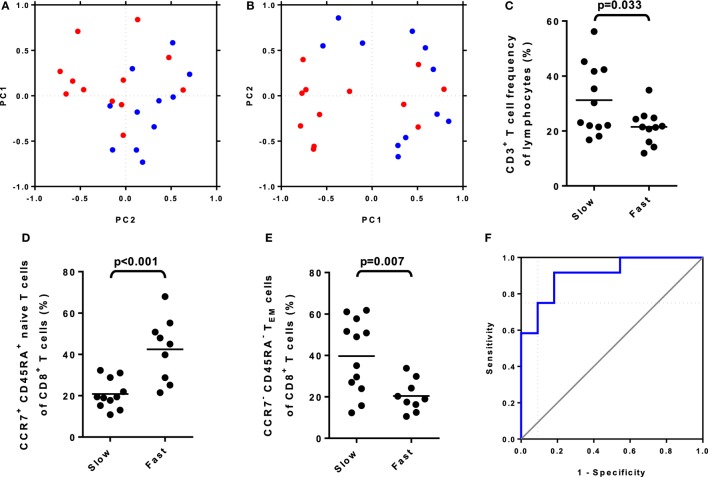
Statistical analyses of flow cytometry dataset. **(A)** The first two principal components (PC1, PC2) of entire dataset of baseline standardized leukocyte frequencies partly distinguished fast (red) and slow (blue) responders. **(B)** After filtering the baseline dataset for leukocyte populations whose frequencies were significantly influenced by direct-acting antiviral treatments, the first two principal components of standardized leukocyte frequencies separated fast (red) and slow (blue) responders. **(C)** Comparison of baseline CD3^+^ T cell frequency between fast and slow responders. **(D)** Comparison of baseline naïve CD8^+^ T cell frequency between fast and slow responders. **(E)** Comparison of baseline CD8^+^ T_EM_ cell frequency between fast and slow responders. **(F)** Baseline CD3^+^ T cell frequency and baseline naïve CD8^+^ T cell frequency were entered as independent variables in a binary logistic regression model. A cutoff probability of 0.66 for scoring fast and slow responders was determined using receiver operator characteristic curve analysis, which gave a test sensitivity of 75.0% and specificity of 91.0%.

We first asked whether the baseline data contained enough information to distinguish fast and slow responders. By standardizing the data and reducing their complexity by PCA, partial separation of fast- and slow-responding patients was possible (Figure 1A). We next asked whether a set of baseline immunological parameters could be identified that distinguished patients as fast or slow responders. To restrict the number of independent variables in our models, we considered only parameters that changed significantly between pre- and posttreatment samples (Supplement S4 in Supplementary Material). This approach was based on the assumption that changes in immunological parameters after DAA treatment were likely to reflect changes in the immune response against the virus or secondary effects of changes in antiviral immunity. Our group has previously reported that biologically relevant changes in leukocyte subset frequencies in individual patients may be relatively small compared to biological variation between individuals; therefore, comparing baseline-subtracted values between serial samples can be a more sensitive way to detect individual immunological changes over time than comparing simple frequencies (16). Changes in baseline-subtracted leukocyte subset frequencies between visits were identified by pairwise significance testing. After starting DAA treatment, an increase was observed in the frequency of CD8^+^ T cells, and CD4^+^ and CD8^+^ central memory T cells (T_CM_); by contrast, a decrease was observed in the frequency of CD4^+^ and CD8^+^ effector memory T cells (T_EM_), CD4^+^ T_EMRA_ cells, naïve CD4^+^ T cells, non-classical CD14^+^CD16^+^ monocytes and CD56^bright^ NK cells. Filtering the dataset for these significantly changed variables improved separation of fast and slow responders by PCA (Figure 1B).

### A Model to Predict Fast and Slow Responses

Univariate analyses were performed to determine which of those parameters affected by DAA treatment were most significantly associated with fast or slow responder status (Figures 1C–E). It is a generally accepted rule-of-thumb that a minimum of 10 events per variable represents adequate sample size for regression analyses (17). The frequencies of CD3^+^ T cells with respect to CD45^+^ leukocytes and CCR7^+^ CD45RA^+^ naïve CD8^+^ T cells with respect to CD8^+^ T cells were entered into a binary logistic regression model as the two most significant and biologically non-redundant independent variables.

Receiver operator characteristic curve analysis (area = 0.909; SE = 0.61) was used to determine a cutoff value of the predicted probabilities that maximized both sensitivity (75.0%) and specificity (91.0%) (Figure 1F). Using a cutoff value of 0.66, pretreatment frequencies of CD3^+^ T cells and CCR7^+^ CD45RA^+^ naïve CD8^+^ T cells correctly classified 82.6% of patients as fast or slow responders (compared to 52.2% with no model, i.e., classifying all patients as fast responders) in our study cohort (Table 2A,B). Notably, the 95% confidence intervals (CIs) of the odds ratios were narrow and the model was a good fit (Nagelkerke R-squared = 0.623).

**Table 2:** Logistic regression analysis of baseline leukocyte frequencies and classification of patients according to the predicted cut-off.

(A) Logistic regression analysis				
Parameter		Odds ratio (95% CI)		*p*-value
CCR7^+^ CD45RA^+^ naïve CD8^+^ T cells		1.126 (1.000, 1.269)		0.051
CD3^+^ T cells		0.862 (0.729, 1.021)		0.087

**(B) Classification table^a^: all patients**				

**Predicted**		**Observed**		
		**Response**		**Percentage (point estimate, 95% CI)**
		**Slow**	**Fast**	

Response	Slow	10	3	76.9 (46–95)
	Fast	1	9	90.0 (55–100)
Overall percentage				82.6

**(C) Classification table^a^: patients with fibrosis score 1–4**				

**Predicted**		**Observed**		
		**Response**		**Percentage (point estimate, 95% CI)**
		**Slow**	**Fast**	

Response	Slow	9	2	81.8 (48–98)
	Fast	0	4	100.0 (28–100)
Overall percentage				86.7

**(D) Classification table^a^: prospective set**				

**Predicted**		**Observed**		
		**Response**		**Percentage (point estimate, 95% CI)**
		**Slow**	**Fast**	

Response	Slow	8	1	88.9 (52–100)
	Fast	0	1	100.0 (1–100)
Overall percentage				90.0

*(A) Odds ratios and 95% confidence intervals. (B) Classification of 23 study patients as either fast or slow responders to direct-acting antiviral (DAA) treatment according to only baseline CD3^+^ T cell and CCR7^+^ CD45RA^+^ naïve CD8^+^ T cell frequencies. (C) Classification of 15 study patients with hepatic fibrosis as either fast or slow responders to DAA treatment according to only baseline CD3^+^ T cell and CCR7^+^ CD45RA^+^ naïve CD8^+^ T cell frequencies. (D) Prospective classification of 10 patients*.

*^a^Cutoff value is 0.66*.

Baseline score for liver fibrosis was not associated with responder status (Supplement S3 in Supplementary Material). Therefore, we asked how well our immunological markers classified the 15/23 study patients with any degree of liver fibrosis (Table 2C). The overall correct classification rate was 86.6%, compared to 60.0% correct classification under the null assumption that all patients with liver fibrosis were slow responders. Importantly, the true positive rate for fast responders with liver fibrosis was 4/4 (100%) patients; accordingly, we believe that our assay will be particularly useful in identifying which patients with chronic HCV infection and liver fibrosis will be fast responders to DAA treatment.

To assess whether our model correctly predicted fast and slow responses, data were prospectively collected from a further 10 chronic HCV patients prior to DAA treatment (Supplement S5 in Supplementary Material). A formal external validation of our model, which would typically require a sample size of more than 100 events, was beyond the scope of this study (18). In this prospective group, eight patients were slow responders and two were fast responders: all slow responders were corrected classified as such (Table 2D). Our model correctly identified one of two fast responders. For the purpose of prospectively and safely shortening DAA therapy, a test with high positive predictive value (PPV) is more important than one with high sensitivity. Importantly, the only patient predicted to be a fast responder proved to be so.

### Mechanisms Underlying the Predictive Model

**Figure 2:**
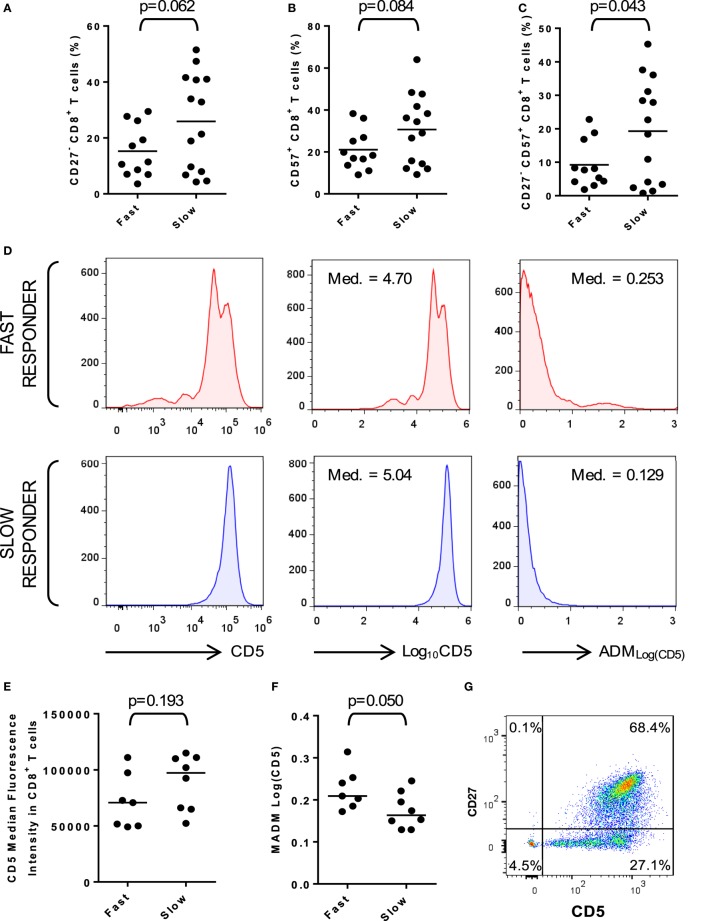
Generalized activation of peripheral blood CD8^+^ T cells in slow responders. **(A)** Frequencies of CD27^−^ CD8^+^ T cells in fast and slow responders. **(B)** Frequencies of CD57^+^ CD8^+^ T cells in fast and slow responders. **(C)** Frequencies of chronically activated CD27^−^ CD57^+^ CD8^+^ T cells in fast and slow responders. **(D)** Visual representation of the method used to estimate the dispersion of CD5 expression in CD8^+^ T cells. The examples represent one fast and one slow responder from samples of *n* = 7 and *n* = 8, respectively. The first pair of histograms show CD5 fluorescence intensities of >1 plotted on a log-axis. The second pair of histograms show log_10_-transformed values plotted on a linear-axis and their respective medians (Med.). The third pair of histograms show absolute deviation of log_10_-transformed values from median (ADM) and the respective medians (MADM). **(E)** CD5 expression by CD8^+^ T cells from fast and slow responders estimated by median fluorescence intensity. **(F)** MADM_Log(CD5)_ in CD8^+^ T cells from fast and slow responders. Assessing the spread of CD5 expression in CD8^+^ T cells discriminates better between fast and slow responders. **(G)** Downregulation of CD5 expression appears to be confined to CD27^−^ CD8^+^ T cells.

Why should a predictive model based on memory T cell subset distribution correctly classify fast or slow responders to DAA therapy when the drug’s action does not apparently rely on immunological mechanisms? To address this question, we reexamined combined results from the training and prospective datasets. Because slow responders were characterized by higher frequencies of CD3^+^ T cells and lower frequencies of naïve CD8^+^ T cells, our reanalysis focused on markers of CD8^+^ T cell activation and differentiation. The most prominent finding was an over-representation of CD27^−^ CD57^+^ CD8^+^ T cells in slow responders (Figures 2A–C) implying a relative accumulation of chronically activated CD8^+^ T cells in those individuals. These activated T cells are vastly too frequent to be virus-specific (19); instead, their relative abundance is likely to be an indirect consequence of chronic HCV infection. Others have shown that HCV-specific, T cell-mediated responses enhance “bystander” activation of virus-non-specific T cells through release of inflammatory mediators, which systemically alters CD5 expression by naïve CD8^+^ T cells, thereby lowering their activation threshold (19). We hypothesize that immunological parameters used in our predictive model are surrogate markers of an HCV-specific response, which would otherwise be extremely difficult to measure with routine clinical diagnostic assays. Initially, our finding that slow responders exhibited *higher* frequencies of activated and memory phenotype CD8^+^ T cells than fast responders might seem to contradict this hypothesis. However, the relationship between effectiveness or chronicity of specific HCV responses and the magnitude of bystander CD8^+^ T cell activation is not understood; therefore, our observations might be explained by greater or more chronic, but *less effective* virus-specific responses cumulating in greater bystander hyperactivation over time. In support of this explanation, fast responders exhibited a significantly broader spread of CD5 expression in CD8^+^ T cells than slow responders (Figures 2D–F). CD5 downregulation was confined to CD27^−^ CD8^+^ T cells (Figure 2G).

### A Consolidated Assay

**Figure 3:**
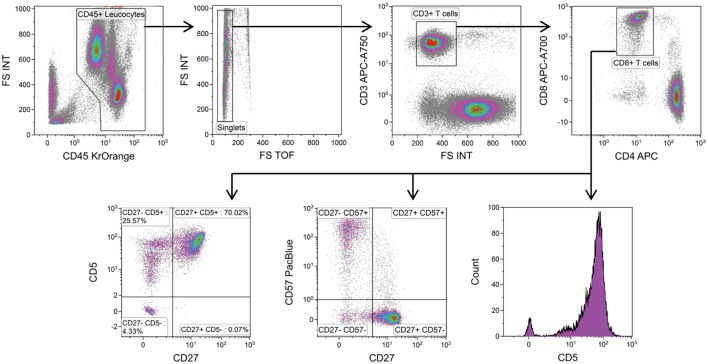
A consolidated flow cytometry panel and recommended gating strategy. Example data from one patient with chronic hepatitis C virus infection is shown.

In order to simplify and standardize our analytical methods, we created a single, 10-color flow cytometry panel to measure frequencies of CD3^+^ T cells, CD45RA^+^ CCR7^+^ CD8^+^ naïve T cells, CD27^−^ CD57^+^ CD8^+^ T cells, and CD5-expressing CD8^+^ T cells (Table 1). In this panel, one channel is available for live/dead cell discrimination using 7-aminoactinomycin D (7-AAD). Fresh material is typically analyzed without 7-AAD staining, whereas analysis of stored material always requires live/dead cell discrimination. Absolute quantification of CD5 expression is possible using fluorescence calibration beads. Populations of interest are clearly resolved and gating is intuitive (Figure 3).

## Discussion

This study took an unbiased approach to biomarker discovery by screening chronic HCV patients receiving DAA treatment for changes in diverse peripheral blood leukocyte populations, including subsets of T cells, B cells, NK cells, monocytes, and blood dendritic cells (DCs). Prominent, but focused changes in the frequencies of non-classical monocytes, CD56^bright^ NK cells and memory T cell subsets were associated with viral clearance after DAA treatment; however, no definite effects were found in B cell, classical NK cell, and blood DC subsets.

The relatively narrow immunological impact of DAA treatment is convenient when building a predictive model because fewer independent variables need to be considered. We found that knowing only pretreatment frequencies of CD3^+^ T cells and CCR7^+^ CD45RA^+^ naïve CD8^+^ T cells allowed 82.6% of patients to be correctly classified as fast or slow responders. Importantly, a true positive prediction rate of 90.0% means that our classifier performs very well in identifying patients as fast responders. A method of reliably identifying fast responders opens the possibility of treating them with shorter courses of DAA therapy, thereby cutting costs to healthcare providers. Current EASL guidelines recommend that a small subgroup of treatment-naï ve patients without cirrhosis and with GT1 infection can be treated for only 8 weeks if their baseline HCV RNA level is below 6 million international units per milliliter (8). We cautiously suggest that our classifier might eventually be used to extend this recommendation to predicted fast responders; naturally, this could have a very significant economic impact. In a recent proof-of-concept study, Lau and colleagues were able to define a subset of patients with chronic HCV GT 1b infection and no cirrhosis who made “ultrarapid” virological responses on triple DAA regimens and could be cured within only 3 weeks (20). The role of on treatment HCV RNA testing for SVR prediction has diminished since approval of IFN-free DAA regimens. However, Maasoumy and Vermehren recently showed that HCV RNA load at week 4 is highly predictive (PPV = 82%) of SVR in GT3 patients on SOF plus RBV therapy (4). Furthermore, the rate of early viral clearance in chronic HCV patients treated with SOF in combination with simeprevir, DCV, or LDV has been correlated with time to viral clearance (21). Although response-guided therapy is currently not recommended for any approved DAA agents, future attempts at individualization of treatment duration may still be beneficial considering the high costs of therapy. We suggest that our approach to predicting DAA responses prior to starting therapy could complement future treatment algorithms, especially in difficult-to-treat patients.

The striking association between fast or slow responder status and altered distribution of T cell subsets raises the question of a causal relationship. Slow responders were characterized by (1) a higher frequency of CD3^+^ T cells within the CD45^+^ leukocyte pool, (2) a higher proportion of T_EM_ cells in the CD8^+^ T cell compartment, and (3) a higher proportion of CD27^−^ CD57^+^ CD8^+^ T cells. These overrepresented subsets of CD8^+^ T cells were vastly too frequent to represent an expansion of HCV-specific T cells (19). There was no evidence for functional impairment of these T cells, either in terms of their ability to secrete IFN-γ or expression of exhaustion markers, including CD49b, CD223, CD279, CD160, and CD244 (data not shown). Therefore, consistent with earlier reports, our results point toward systemic activation of non-specific CD8^+^ T cell responses through a bystander mechanism. We speculate that association between prolonged time-to-clearance of virus and higher frequencies of chronically activated T cells is a reflection of greater or more chronic, but ultimately less effective virus-specific responses in slow-responding individuals. It is noted that 22 of 23 training set patients and 10 of 10 prospective set patients achieved SVR12; therefore, the immunological phenomena discussed here are not general determinants of success with DAA therapy, but are predictive of time to viral clearance. In future studies, we plan to explore possible relationships between exaggerated bystander hyperactivation and outright treatment failure in difficult-to-treat patients.

Potential reasons for DAA treatment failure have been described by other investigators and can be broadly categorized as host-, virus-, or treatment-related factors. At this time, we suspect that the immunological profile associated with fast or slow response to DAA treatment is driven by a specific reaction to HCV virus itself; however, it is unclear whether our classifier is also affected by other consequences of HCV infection, such as hepatic fibrosis. Taken alone, a variety of conventional parameters (i.e., pretreatment ALT levels, viral GT, patient age, previous HCV treatment, and baseline fibrosis score) did not classify patients as fast or slow responders with the same accuracy as our two-parameter model. It is a limitation of this study that the number of cases did not allow more independent variables to be included in our model; nevertheless, in the future, our predictions could be further refined by including additional immunological, clinical, virological, or histopathological parameters.

What is the likely clinical impact of this study? Considering the very high cost of DAA therapy, strategies for reliably individualizing treatment duration could have very substantial cost implications. Flow cytometry is a widely available diagnostic modality in Clinical Chemistry laboratories, where it is used for diagnosis of hematological and immunological disorders. The flow cytometry-based assays used for this study are highly standardized and the required reagents are commercially available. The assays can be performed for under $100 in less than 4 h. Revalidation of our predictive model is currently underway using our consolidated flow cytometry panel, which should lead to further reduced costs and processing time. Based on a recent study, every patient given 6–10 weeks’ instead of 12 weeks’ treatment would save up to $50,000 on the cost of DAA’s (21). Therefore, economically at least, there is a strong case for using our classifier as a guide to more rationalized prescribing of DAA treatment. Of course, such cost considerations must be set against the surety and convenience of one-size-fits-all regimens.

In summary, the pretreatment frequencies of chronically activated CD8^+^ T cells and subsets of memory CD8^+^ T cells can be used to reliably predict early viral control in patients receiving DAA therapy for chronic HCV infection. In the future, this discovery could open the possibility of shortening DAA therapy in individuals predicted to be fast responders, thereby substantially reducing costs.

## Ethics Statement

Peripheral blood samples were provided by patients with chronic HCV infection, who were participating in an observational trial (clinicaltrials.gov: NCT02904603) authorized by the local ethics committee (votum: 14-101-0049). From all participating patients, an informed written consent was obtained.

## Author Contributions

Conception and design of the study: JAH, KW, HS, EG, and JW. Data acquisition: JAH, AA, JH, PR, and JW. Analysis and interpretation of the data: JAH, FZ, MH, NA, HS, EG, and JW. Statistical analysis: FZ and JAH. Drafting of the manuscript: JAH and JW. Revision of the manuscript: JAH, EG, and JW. All authors had access to the study data and critically reviewed and approved the final version of the manuscript.

## Conflict of Interest Statement

The authors declare that no competing interests exist. HS and KW receive consulting and lecture fees. The remaining authors receive no external income.
