# Supplementary material for: Predicting Early Viral Control under Direct-Acting Antiviral Therapy for Chronic Hepatitis C Virus Using Pretreatment Immunological Markers
Source: Front Immunol. 2018 Feb 7;9:146. doi: 10.3389/fimmu.2018.00146 (PMC5808305; doi:10.3389/fimmu.2018.00146)
Supplement: Supplementary file 1 [file Data_Sheet_1.DOCX]

**Supplementary materials**

**Suppl.1** Table of patient characteristics

**Suppl.2** Summary of clinical outcomes

**Suppl.3** Association of pre-treatment ALT levels, HCV RNA titre and fibrosis score with fast or slow responder status.

**Suppl.4** A graphical summary of significant changes in leucocyte subset frequency between baseline and subsequent visits.

**Suppl.5** Table of prospective-set patient characteristics and outcomes

**Supplementary material 1**

Table of patient characteristics

**Supplementary material 2**

Table of patient outcomes


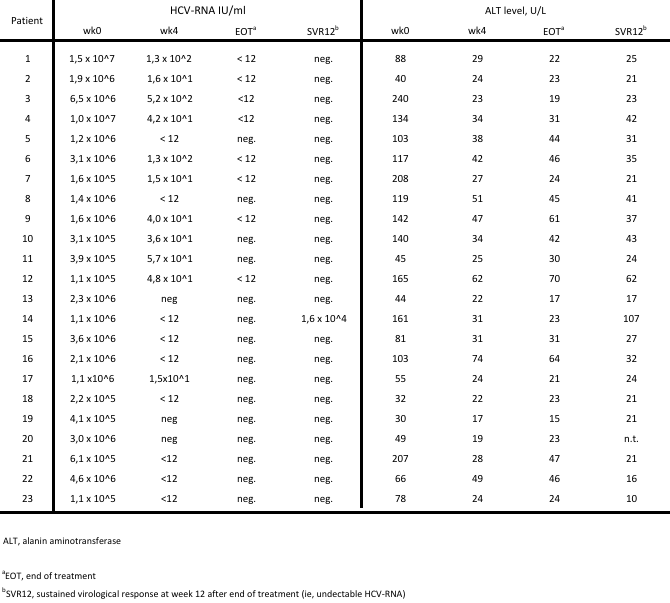


**Supplementary material 3**

**Pre-treatment ALT levels**

Baseline ALT levels were not associated with fast or slow responses.

|  |  | ALT level (U/L) | | |
| --- | --- | --- | --- | --- |
|  |  | <50 | 50-150 | >150 |
| Response | Fast | 4 | 6 | 2 |
|  | Slow | 2 | 6 | 3 |

*Χ*^2^ test: p = 0.662

**HCV Genotype**

HCV genotype was not associated with fast or slow responses.

|  |  | HCV Genotype | | |
| --- | --- | --- | --- | --- |
|  |  | 1a | 1b | 3a |
| Response | Fast | 5 | 4 | 3 |
|  | Slow | 5 | 5 | 1 |

*Χ*^2^ test: p = 0.586

**Previous treatment for chronic HCV**

Patients were categorised as having previously been treated with PegIFN/RBV-based therapies or not. Previous treatment was not significantly associated with fast or slow responses to DAA treatment.

|  |  | Previous Treatment | |
| --- | --- | --- | --- |
|  |  | Yes | No |
| Observed | Fast | 7 | 5 |
|  | Slow | 4 | 7 |

*Χ*^2^ test: p = 0.292

**Pre-treatment fibrosis score**

Absence of liver fibrosis at baseline was not associated with fast or slow responses.

|  |  | Fibrosis Score | | | | |
| --- | --- | --- | --- | --- | --- | --- |
|  |  | F0 | F1 | F2 | F3 | F4 |
| Response | Fast | 6 | 2 | 1 | 1 | 2 |
|  | Slow | 2 | 2 | 1 | 2 | 4 |

*Χ*^2^ test: p = 0.564

To further test the capacity of baseline fibrosis score to correctly classify patients as fast or slow responders, the table was collapsed as follows:

*Grouping 1*:

|  |  | Predicted | |
| --- | --- | --- | --- |
|  |  | Fast (F0) | Slow (F1 - F4) |
| Observed | Fast | 6 | 6 |
|  | Slow | 2 | 9 |

*Χ*^2^ test: p = 0.110; correct classification rate = 65.2%

*Grouping 2*:

|  |  | Predicted | |
| --- | --- | --- | --- |
|  |  | Fast (F0 - F1) | Slow (F2 - F4) |
| Observed | Fast | 8 | 4 |
|  | Slow | 4 | 7 |

*Χ*^2^ test: p = 0.146; correct classification rate = 65.2%

These data show that absence of fibrosis (or low-grade fibrosis) is not associated with being a fast responder.

**Patient age**

Changes in memory T cell subset distribution have been associated with ageing; therefore, association between patient age and responder status was examined.

Training Set

|  | 20-29 years | 30-39 years | 40-49 years | 50-59 years | 60-69 years | 70+ years |
| --- | --- | --- | --- | --- | --- | --- |
| SLOW | 0 | 0 | 2 | 2 | 5 | 2 |
| FAST | 1 | 1 | 3 | 3 | 2 | 2 |

*Χ*^2^ test: p = 0.601

Validation Set

|  | 20-29 years | 30-39 years | 40-49 years | 50-59 years | 60-69 years | 70+ years |
| --- | --- | --- | --- | --- | --- | --- |
| SLOW | 1 | 0 | 0 | 3 | 3 | 1 |
| FAST | 0 | 0 | 1 | 0 | 0 | 1 |

*Χ*^2^ test: p = 0.143

Combined Training and Validation Set

|  | 20-29 years | 30-39 years | 40-49 years | 50-59 years | 60-69 years | 70+ years |
| --- | --- | --- | --- | --- | --- | --- |
| SLOW | 1 | 0 | 2 | 5 | 8 | 3 |
| FAST | 1 | 1 | 4 | 3 | 2 | 3 |

*Χ*^2^ test: p = 0.401

No association between patient age and responder status was observed.

**Supplementary material 4**

A graphical summary of significant changes in leucocyte subset frequency between baseline and subsequent visits. Because biologically relevant changes in leucocyte subset frequencies in individual patients may be relatively small compared to biological variation between individuals, comparing individual baseline-subtracted values between serial samples can be a sensitive approach for detecting immunological changes. Significant changes in baseline-subtracted leucocyte subset frequencies between visits were identified by pairwise significance testing (n=23).

**Supplementary material 5**

Table of prospective-set patient characteristics and outcome
